# Endothelial deletion of the cytochrome P450 reductase leads to cardiac remodelling

**DOI:** 10.3389/fphys.2022.1056369

**Published:** 2022-12-02

**Authors:** Melina Lopez, Pedro F. Malacarne, Deepak P. Ramanujam, Timothy Warwick, Niklas Müller, Jiong Hu, Matthias Dewenter, Andreas Weigert, Stefan Günther, Ralf Gilsbach, Stefan Engelhardt, Ralf P. Brandes, Flávia Rezende

**Affiliations:** ^1^ Institute for Cardiovascular Physiology, Goethe-University, Frankfurt, Germany; ^2^ German Center of Cardiovascular Research (DZHK), Frankfurt, Germany; ^3^ Institute of Pharmacology and Toxicology, Technical University, Munich, Germany; ^4^ German Center of Cardiovascular Research (DZHK), Munich, Germany; ^5^ Institute for Vascular Signaling, Goethe-University, Frankfurt, Germany; ^6^ Institute of Experimental Cardiology, Heidelberg, Germany; ^7^ Institute of Biochemistry I, Goethe University, Frankfurt, Germany; ^8^ Max-Planck-Institute for Heart and Lung Research, Bad Nauheim, Germany

**Keywords:** cytochrome P450 reductase, cardiac remodelling, echocardiography, endothelial cells, cardiac myocytes, transverse aortic constriction

## Abstract

The cytochrome P450 reductase (POR) transfers electrons to all microsomal cytochrome P450 enzymes (CYP450) thereby driving their activity. In the vascular system, the POR/CYP450 system has been linked to the production of epoxyeicosatrienoic acids (EETs) but also to the generation of reactive oxygen species. In cardiac myocytes (CMs), EETs have been shown to modulate the cardiac function and have cardioprotective effects. The functional importance of the endothelial POR/CYP450 system in the heart is unclear and was studied here using endothelial cell-specific, inducible knockout mice of POR (ecPOR^−/−^). RNA sequencing of murine cardiac cells revealed a cell type-specific expression of different CYP450 homologues. Cardiac endothelial cells mainly expressed members of the CYP2 family which produces EETs, and of the CYP4 family that generates omega fatty acids. Tamoxifen-induced endothelial deletion of POR in mice led to cardiac remodelling under basal conditions, as shown by an increase in heart weight to body weight ratio and an increased CM area as compared to control animals. Endothelial deletion of POR was associated with a significant increase in endothelial genes linked to protein synthesis with no changes in genes of the oxidative stress response. CM of ecPOR^−/−^ mice exhibited attenuated expression of genes linked to mitochondrial function and an increase in genes related to cardiac myocyte contractility. In a model of pressure overload (transverse aortic constriction, TAC with O-rings), ecPOR^−/−^ mice exhibited an accelerated reduction in cardiac output (CO) and stroke volume (SV) as compared to control mice. These results suggest that loss of endothelial POR along with a reduction in EETs leads to an increase in vascular stiffness and loss in cardioprotection, resulting in cardiac remodelling.

## 1 Introduction

Different cell types constitute the heart making it a complex multicellular organ with an integrated network of intercellular communication (Perbellini et al., 2018; Kivelä et al., 2019). Although cardiac myocytes (CM) are the predominant contributor to heart mass, the much small endothelial cells (EC), which form the cardiac capillaries, are the most abundant cell type of the heart (Kivelä et al., 2019). The cardiac microcirculation supplies cardiac myocytes with nutrients and oxygen. Cardiac hypertrophy therefore requires an expansion of the microcirculation and transition from hypertrophy to heart failure can be a consequence of an insufficient angiogenic response (Kivelä et al., 2019). In addition to their function as vascular barrier, ECs release autacoids and metabolites. Although it is known that these secreted molecules affect cardiac myocyte function, the effect induced by individual signals and their pathophysiological relevance are still not clear (Talman and Kivelä, 2018; Kivelä et al., 2019).

The cytochrome P450 (CYP) system is a superfamily of membrane-bound monooxygenases which facilitates, among other functions, metabolism of fatty acids and production of vasoactive lipids, i.e., epoxyeicosatrienoic acids (EETs) (Hu et al., 2017; Wang et al., 2021; Frömel et al., 2022). The activity of all microsomal CYP enzymes depends on the cytochrome P450 reductase (POR) which transfers electrons from NADPH through FAD/FMN domains to the heme within the CYP450 enzymes (Jensen et al., 2021). In the catalytic cycle of POR/CYP450, the substrate first binds to a ferric CYP450 (heme-bound) forming a complex which binds to oxygen and accepts electrons from POR. Oxygen becomes activated at the heme centre of CYP450 and thereby the substrate can be hydroxylated (R-OH). During these electron transfers, reactive oxygen species (ROS) can be released as a side product owing to the decay of the one-electron-reduced ternary complex (Pandey and Flück, 2013). Thus, POR releases a fraction of activated oxygen from the enzyme without substrate oxidation, making the POR/CYP450 system a potential source of ROS (Sun et al., 2012).

Although the liver expresses the highest level of CYP450 isoforms; extrahepatic expression is not negligible (Fleming, 2001; Chaudhary et al., 2009; Wang et al., 2021). Vascular cells have been shown to express the CYP2C and CYP2J epoxygenases as well as the CYP4A ω-hydroxylase (Fleming 2014). CYP2C and CYP2J generate EETs in EC, whereas CYP4 produces the vasoconstrictor 20-hydroxyeicosarrienoic acid (20-HETE) in vascular smooth muscle cells (VSMCs) (Fleming, 2014) but also hydroxylate long chain fatty acids which are further converted to dicarboxylic acids to fuel β-oxidation for energy production (Hardwick, 2008). Endothelial-derived EETs act as endothelium-dependent vasodilator in some mammals (Cazade et al., 2014; Fleming, 2014). Additionally, EETs have anti-inflammatory properties and can mediate cardioprotection during myocardial ischemia-reperfusion. Whether the latter function is mediated by cardiac myocyte-derived EETs only or also through endothelial cells is unclear (Oni-Orisan et al., 2014).

As mentioned above, CYP450 enzymes require POR for their activity. POR is coded by a single gene only, whereas the microsomal CYP450 isoenzymes are coded by 90 different genes in mice (Zanger and Schwab, 2013). Deletion of POR therefore is a powerful approach to inactivate all CYP450 isoenzymes. In order to identify the cardiac relevance of endothelial CYP450 isoenzymes we generated an endothelial-specific inducible POR knockout mouse (ecPOR^−/−^) and characterized the functional consequences for the heart.

## 2 Material and methods

### 2.1 Chemicals

All chemicals were purchased from Sigma-Aldrich (St. Louis, Missouri, United States) unless otherwise indicated.

### 2.2 Animal procedure

Endothelial cell-specific, tamoxifen inducible POR knockout mice (ecPOR^−/−^) were generated as previously described (Malacarne et al., 2022). Briefly, Por^flox/flox^ mice (Por^tm1Ding^) (Wu et al., 2003) were crossed with Cdh5-CreERT2 (Tg(Cdh5-CreERT2)^1Rha^) (Wang et al., 2010) mice. POR deletion was induced by tamoxifen feeding (CreActive TAM400, #D.T400.R1, Laval, France, 1 mg/mouse/day, 10 days) when mice were at least 8 weeks old followed by a “wash-out period” of at least 30 days. Por^flox/flox^-Cdh5-CreERT2^0/0^ littermates (no cre expression) with tamoxifen treatment served as control (CTL). Animals were housed in groups with free access to chow and water in a specified pathogen-free facility with a 12h-day/12h-night cycle. All animal experiments were performed in accordance with the German animal protection law and were carried out after approval by the local authorities (FU1188). Unless specified, all animal experiments were performed 30 days after inducing the knockout.

### 2.3 Histology

Left ventricles (LV) from ecPOR^−/−^ mice and control littermates were freshly isolated, embedded in OCT^TM^ (Tissue-Tek^®^) and frozen on dry ice. Frozen tissues were cut (8 µm) over Superfrost^®^ slides. For assessment of the cross-sectional area of cardiac myocytes, heart sections were stained with wheat germ agglutinin (WGA, Alexa Fluor 647 conjugate, #W32466, dilution 1:100, Life Technologies, Carlsbad, United States). For this, sections were immersed in an immersion chamber filled with sodium-citrate buffer, heated for 4 min in the microwave and cooled at RT. Sections were then incubated for 1.5 h with Alexa-Fluor 647-WGA. Nuclei were stained with SYTOX Green (#S7020, dilution 1:1000, Life Technologies, Carlsbad, United States). Images were taken from areas of transversely cut muscle fibers using a confocal microscope (Leica TCS SP5 II). The average cross-sectional area of cardiomyocytes was determined using the Metamorph software (Ramanujam et al., 2021).

### 2.4 Analysis of CYP450 isoenzymes from published sequencing data

The expression of CYP450 isoenzymes in the heart of healthy mice (8–10 weeks old) was profiled using a previously published data set (Ramanujam et al., 2016; Hinkel et al., 2020) which consists of bulk RNA sequencing from different FACS-sorted cells of heart. Heatmaps were generated with R using the pheatmap package which uses the average expressions (calculated from 3 replicates per cell type) obtained from bulk transcriptome of the sorted cells at baseline. Pheatmap calculated the Z-score automatically for every gene using the below formula:

Z−score=(x−mean(x))/stdev(x).

### 2.5 Isolation of cardiac endothelial cells

Cardiac endothelial cells were isolated from LV of ecPOR^−/−^ and CTL mice using magnetic activated cell sorting. The remaining myocardial tissue was designated as cardiac fraction. Briefly, two LVs per genotype were pooled in Hank’s balanced salt solution (HBSS, 1 ml) and cut into small pieces (1 mm). Minced tissue was transferred to 50 ml Falcon tubes containing enzyme mix (10 ml Dulbecco’s modified Eagle’s (DMEM, Gibco Life Technologies, Carlsbad, United States) containing dispase (1.2 U/ml, #39307800, Roche Diagnostics, Mannheim, Germany), collagenase II (2 mg/ml, #LS004177, Worthington, Lakewood, US), elastase (0.03 mg/ml, #2292, Worthington, Lakewood, US) and digested for 20 min at 37°C. Digestion was stopped with DMEM containing 10% FCS. Undigested tissue was removed by filtration through strainers (100 μm and 70 µm). Cells were spun down (10 min, 500 xg, 4°C) and the pellet was resuspended in 10 ml RBC (redo blood cells) lysis buffer (J62150, Alfa Aesar, Kandel, Germany).

#### 2.5.1 Western blotting of endothelial cells

To remove RBC lysis buffer, samples were centrifuged (5 min, 500 xg, 4°C) and the cell pellet was resuspended in washing buffer (HBSS, 0.5% BSA and 2 mM EDTA, 1 ml) and centrifuged (10 min, 300 xg, 4°C). CD144 antibody (2 μl, BD Biosciences Cat# 555289, RRID:AB_395707) was previously coupled to 50 µl Dynabeads sheep anti-rat IgG (#11035, Thermo Fisher, Darmstadt, Germany). The antibody-coupled beads were added to cells and rotated for 40 min at RT. Samples were place on a magnetic rack and the beads were washed three times in DMEM plus 10% FCS. Cells were eluted from the beads by adding lysis buffer containing (pH 7.4, concentrations in mmol/L): Tris-HCl (50), NaCl (150), sodium pyrophosphate (10), sodiumfluoride (20), Triton x-100 (1%), sodiumdesoxycholate (0.5%), proteinase inhibitor mix, phenylmethylsulfonyl fluoride (1), orthovanadate (2), okadaic acid (0.00001). The cardiac fraction was grinded with TissueLyser LT (Qiagen, Hilden, Germany) in Triton x-100 lysis buffer (300 μl, as described above). After lysis, cardiac fraction was centrifuged (10 min, 13,000 rpm, 4°C) and the supernatant was collected for further analysis. Protein concentration was estimated with the Bradford assay and 30 µg were fractionated by SDS/PAGE followed by western blotting. After incubation with primary antibodies, membranes were incubated with fluorescent dye conjugated antibodies from LI-COR biosciences (Bad Homburg, Germany) and analyzed with an infrared-based detection system (LI-COR, Bad Homburg, Germany). The following antibodies were used: POR (Santa Cruz Biotechnology Cat# sc-25270, RRID:AB_627391), eNOS (BD Biosciences Cat# 610297, RRID:AB_397691) and GAPDH (Thermo Fisher Scientific Cat# PA1-16777, RRID:AB_568552).

#### 2.5.2 RNAseq

After RBC lysis, the samples were spun down (5 min, 500 xg, 4°C). The pellet was resuspended in 6.2 ml cold DPBS and transferred to a 15 ml Falcon tube. 1.8 ml Debris Removal Solution (#130-109-398, Miltenyi Biotec, Bergisch Gladbach, Germany) was added and overlaid very gently with 4 ml cold DPBS and centrifuged (10 min, 3,000 xg, 4°C). The two top phases were removed by aspiration and the Falcon tube was filled up again to 15 ml with cold DPBS. Samples were centrifuged (10 min, 3,000 xg, 4°C). Then, the pellet was resuspended in DMEM +10% FCS (200 µl) containing FITC-conjugated lectin (1:100, #L9381-2MG, Sigma). After 20 min incubation, DMEM +10% FCS (500 µl) were added and samples were centrifuged (5 min, 300 xg, 4°C). The supernatant was discarded and cells were resuspended in FACS buffer 1 (DPBS, 0.5% BSA and 2 mM EDTA, 200 µl) containing DRAQ5 (1:200, #424101, BioLegend, Amsterdam, Netherlands). Cells positive for both DRAQ5 and Lectin were selected and FACS-sorted into FACS buffer 2 (DPBS, 10% FCS, 3 mM EDTA and 25 mM HEPES, 300 µl) with BD FACSymphony^TM^ S6 Cell Sorter (BD Biosciences, San Jose, United States). After sorting, cells were centrifuged (5 min, 500 xg, 4°C) and total RNA was isolated using the RNeasy micro kit (Qiagen, Hilden, Germany) combined with on-column DNase digestion (DNase-Free Dnase set, Qiagen). RNA of 2 mice per genotype was pooled. Total RNA from LV (cardiac fraction) of ecPOR^−/−^ and CTL was isolated using the RNeasy fibrous tissue mini kit (Qiagen, Hilden, Germany) combined with on-column DNase digestion (DNase-Free Dnase set, Qiagen) following the manufacturer’s instructions.

### 2.6 Echocardiography

Cardiac parameters were assessed with ultrasonography (Zacchigna et al., 2021) using a Vevo3100 device (Toronto, Canada). Data analysis was performed using the Vevo LAB desktop software. Measurements were obtained from short-axis M-mode images.

### 2.7 RNAseq

RNA and library preparation integrity were verified with LabChip Gx Touch 24 (Perkin Elmer). For the cardiac fraction 4 µg of total RNA was used as input for VAHTS Stranded mRNA-seq Library preparation following manufacture’s protocol (Vazyme). For EC samples RNA was used for SMART-Seq® v4 Ultra® Low Input RNA Kit (Takara Bio). Sequencing was performed on NextSeq2000 instrument (Illumina) with 1 × 72 bp single end setup. The resulting raw reads were assessed for quality, adapter content and duplication rates with FastQC (RRID:SCR_014583) (Andrews, 2010).

The sequencing reads for all samples were quantified against the hg38 transcriptome (obtained from Ensembl using Salmon (v1.5.2); RRID:ZFIN_ZDB-ALT-170801-8) (Patro et al., 2017; Howe et al., 2020). Reads not aligned to the transcriptome were discarded at this point. Differential gene expression analysis was performed using DESeq2 (v1.32.0; RRID:SCR_015687)) in R (v4.1.1; R Project for Statistical Computing (RRID:SCR_001905) (Love et al., 2014; R Core Team, 2015). Raw transcript counts were summed per gene and used in the standard DESeq2 differential gene expression analysis workflow, using a negative binomial test over gene counts in each of the combinations of conditions. Batch information was also included in the contrast formula, and genes with more than 4 zero counts across samples were removed from the analysis. Heatmaps were created using the R package ggplot2 (RRID:SCR_014601) in R.

### 2.8 Reverse transcription and quantitative real-time PCR

Total RNA was extracted with RNeasy fibrous mini kit (#74704, Qiagen) according to manufacturer’s protocol. Briefly, cardiac tissue (left ventricle, 25 mg) was homogenized (TissueLyser LT, Qiagen) in the RLT buffer (provided in the kit) containing β-mercaptoethanol (10%) and RNA was extracted according to the manufacture’s instructions. Synthesis of cDNA was carried out using SuperScript III reverse transcriptase (#12574026, Thermo Fisher Scientific, Massachusetts, United States) and a combination of oligo(dT)23 and randome hexamer primers (Sigma). Quantitative real-time PCR was performed with 2 ng cDNA using iTaq Universal SYBR Green Supermix with ROX as reference dye (#175121, Bio-Rad) in an AriaMx cycler (Agilent Technologies). Mouse target genes were normalized to β-actin expression. Relative expression was calculated using the ∆∆Ct method with AriaMX qPCR software (Agilent). Primers used for amplification are listed in Table 1.

**TABLE 1:** Primer list for RT-qPCR.

Gene	Forward primer (5′-3′)	Reverse primer (5′-3′)
*Myh6*	GCT​GGA​AGA​TGA​GTG​CTC​AGA​G	CCA​GCC​ATC​TCC​TCT​GTT​AGG​T
*Glut4*	GCC​CGG​ACC​CTA​TAC​CCT​AT	GGT​TCC​CCA​TCG​TCA​GAG​C
*Actb*	AGA​TCA​AGA​TCA​TTG​CTC​CTC​CT	ACG​CAG​CTC​AGT​AAC​AGT​CC

### 2.9 Metabolomics of cardiac tissue

Mice were sacrificed and perfused with Hanks buffer. Untargeted global metabolomics from cardiac tissue (50 mg left ventricule) was performed by Metabolon Inc. (Morrisville, NC, United States) using a Waters ACQUITY ultra-performance liquid chromatography (UPLC) and a Thermo Scientific Q-Exactive high resolution/accurate mass spectrometer interfaced with a heated electrospray ion-ization (HESI-II) source and Orbitrap mass analyzer operating at 35,000 mass resolution as previously described (Evans et al., 2009; Bridgewater BR 2014; Al-Khelaifi et al., 2018). Briefly, samples were extracted with methanol to remove the protein fraction. The extract was divided into five fractions: two for analysis by two separate reverse phase (RP)/UPLC-MS/MS methods with positive ion mode electrospray ionization (ESI), one for analysis by RP/UPLC-MS/MS with negative ion mode ESI, one for analysis by hydrophilic interaction chromatography (HILIC)/UPLC-MS/MS with negative ion mode ESI, and one sample as a backup. Raw data were extracted, peak-identified, and quality control-processed by Metabolon (Corey et al., 2012). Compounds were identified by comparison to library entries with over 3,300 commercially available purified standard compounds (Evans et al., 2009). A batch correction was performed by Metabolon. Following log transformation and imputation of missing values, if any, with the minimum observed value for each compound, Mixed Model Contrasts were used to identify biochemicals that differed significantly between experimental groups. Missing values were imputed, and statistically analyzed using log transformed data. *p* values of <0.05 were considered significant. Visualizations and plots of metabolomics data were generated using the ggplot (3.3.5) package in R (4.1.1).

### 2.10 Transverse aortic constriction

Transverse aortic constriction using O-rings was employed as pressure-overload model to induce cardiac hypertrophy/failure in ecPOR^−/−^ and CTL mice. O-rings (0.50 × 0.50, 70 BUNA-N, #R00020-020-70BNB, Apple Rubber, Lancaster, US) were prepared as described previously (Melleby et al., 2018). Mice received metamizole in the drinking water (2 mg/ml) starting 24 h before surgery. Thirty minutes before the procedure, mice received buprenorphine (0.05 mg/kg) and were anesthetized with isoflurane, intubated and ventilated with oxygen. Mice were placed on a heating plate on a supine position and a longitudinal incision (1–1.5 mm) in the skin on the left side of the thorax was made with sharp scissors (#14058-09, FST, Heidelberg, Germany). Refraction of the major and minor pectoral muscles allowed access to the intercostal muscles. The third intercostal muscle was first pierced with extra fine Graefe forceps (#11151-10, FST, Heidelberg, Germany), then expanded with sharp scissors. The O-ring was placed around the transverse aorta between brachiocephalic and left common carotid arteries using a spinal cord hook (#10162-12, FST, Heidelberg, Germany). Lower and upper sutures were tied with a double knot and excess material was cut. The third intercostal muscle incision was closed with two single-sutures using 6-0 prolene (#8889H, Ethicon, Belgium) and covered by pectoral muscles. The skin was closed with sutures using 6-0 prolene. Mice were placed in a cage over a warming plate until awaking. Analgesic therapy was continued for 4 days (metamizole plus twice/day injections of buprenorphine). Deletion of POR was performed 1 week post-Op by tamoxifen injection (i.p., 333 mg/kg, 100 µl dissolved in sunflower oil on three consecutive days) to induce the knockout of POR.

### 2.11 Statistics

Data are given as means ± standard error of mean (SEM), unless otherwise indicated. Calculations were performed with GraphPad Prism 8.0 (RRID:SCR_002798) or R (package ggplot2). In case of multiple testing, Bonferroni or Tukey correction was applied. For multiple group comparisons, ANOVA followed by post hoc testing was performed. ANOVA for repeated measurements was used for time course echocardiography. Individual statistics of unpaired samples of two groups was performed by t-test and if not normally distributed, by Mann-Whitney test. A *p*-value of <0.05 was considered as significant. n indicates the number of individual experiments or animals.

## 3 Results

### 3.1 Cytochrome P450 reductase and the CYP450 cardiac repertoire

**FIGURE 1:**
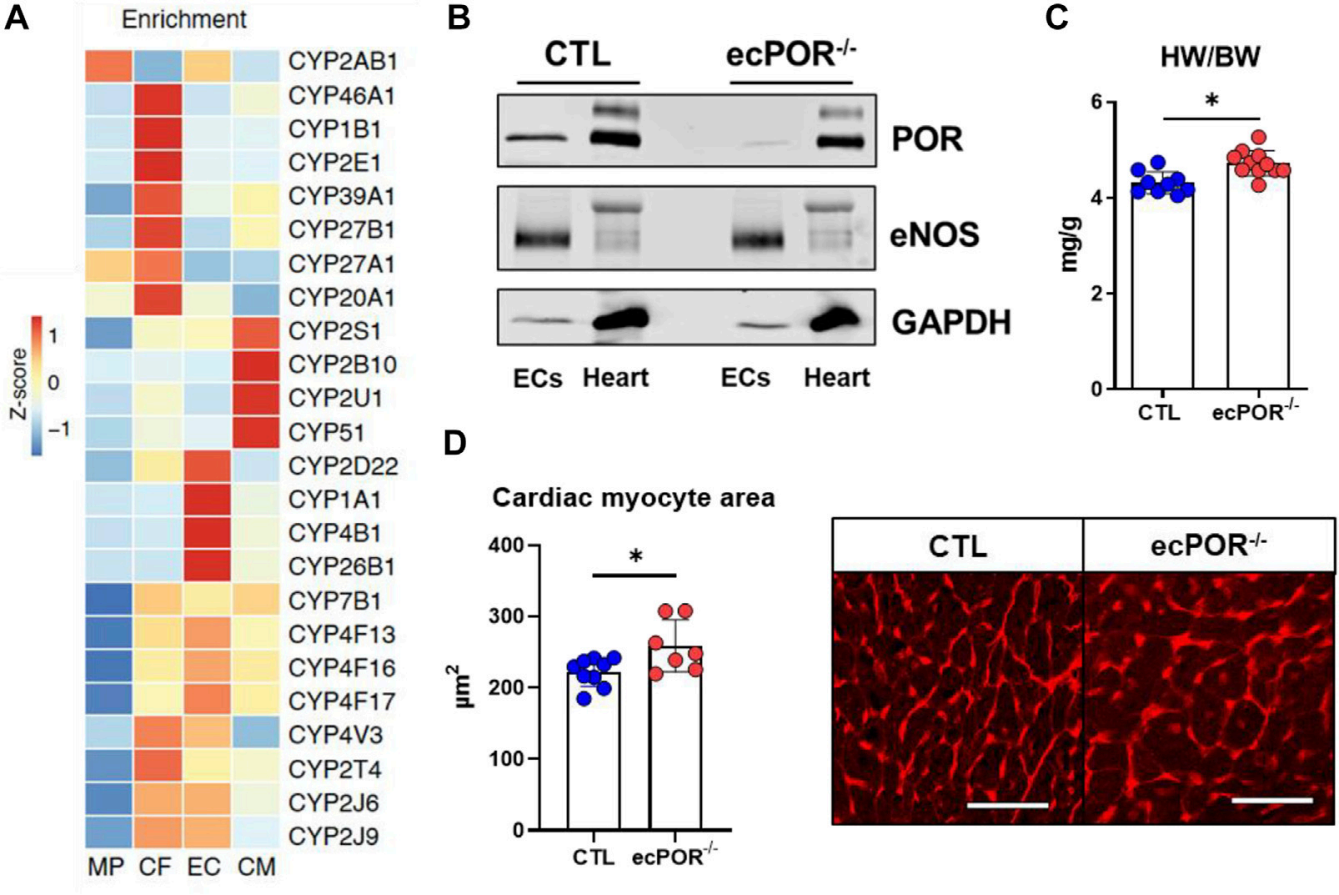
Cytochrome P450 reductase and its cardiac CYP450 repertoire. **(A)** Enrichement of CYP450 genes in different cardiac cell types isolated from healthy murine heart (RNA-sequencing). MP: macrophages, CF: cardiac fibroblasts, EC: endothelial cells, CM: cardiac myocytes. **(B)** Western blotting for POR expression in cardiac endothelial cell (EC) and heart fraction from control (CTL) and ecPOR^−/−^ mice. **(C)** Ratio of heart weight (HW, mg) to body weight (BW, g). n ≥ 11. Body weight CTL: 35.94 ± 2.56; Body weight ecPOR^−/−^: 36.33 ± 2.92. **(D)** Area of cardiac myocyte as determined by Wheat Germ Agglutinin (WGA) staining. n ≥ 7 scale bar: 50 μm *p < 0.05 ecPOR^−/−^ as compared to CTL mice, Mann-Whitney test.

In order to provide a mechanistic basis for a cell type-specific CYP450 function in the heart, CYP450 expression in different cardiac cell type was determined by RNAseq from previously published work (Ramanujam et al., 2016; Hinkel et al., 2020). The cardiac CYP450 expression exhibits cell-type specific expression patterns (Figure 1A). Cardiac endothelial cells (ECs) showed an enriched expression for Cyp1a1 and Cyp4b1, Cyp4f13, Cyp4f16 and Cyp4f17 of the Cyp4 family. This family is known to catalyse the ω-hydroxylation of saturated, branched and unsaturated fatty acids (Hardwick, 2008). CYP450-producing EETs such as Cyp2j6 and Cyp2j9 were expressed at similar level in EC and cardiac fibroblasts (CF). Moreover, CYP450 which metabolize cholesterol (Cyp51) and ω-hydroxylation of AA (Cyp2u1) were mostly expressed in cardiac myocytes (CM). Next, the cardiac CYP450 isoenzymes identified in mice were analysed for their corresponding genes in human as the gene nomenclature differs between the species. A potential genotype-phenotype association was determined with the online tool PhenoScanner (Staley et al., 2016; Kamat et al., 2019) (Table 2). This revealed that only CYP450 enriched in EC exhibited genetic variants associated with diseases and traits related to cholesterol and fatty acid metabolism, congestive heart failure as well as cardiomyopathy.

**TABLE 2:** Genotype-phenotype association obtained with PhenoScanner (http://www.phenoscanner.medschl.cam.ac.uk/) for cardiac CYP450 isoenzymes.

Gene (human)	Gene (mouse)	rsll)	Position	Base	Base	Trait	Type	*p*-value
CYP1A1	Cyplal	rs17861084	chrl5:75O12O19	T	G	Total cholesterol	Diseases and traits	6.14E-1O
		rs17861084	chrl5:75012019	T	G	Triglycerides	Diseases andtraits	121E-07
CYP4B1	Cyp4bl	rs45468297	chrl:47282626	A	G	Cause of death: congestive heart failure	Diseases andtraits	7.36E-13
CYP4F2	Cyp4fl3	rs30931 11	chrl9:1600777O	A	G	LDL cholesterol	Diseases and traits	9.45E-09
		rs3093193	chrl9:15991914	C	G	Coronaryarterydisease	Diseasesandtraits	2.11E-06
CYP4F11	Cyp4fl6	rs144570608	chrl9:16045076	A	T	Cause of death: congestive heart failure	Diseases andtraits	2.82E-16
CYP4F12	Cyp4fl7	s538943818	chrl9:15794653	T	C	Cause of death: dilated cardiomyopathy	Diseases and traits	4.38E-15
		s55O467O38	chrl9:15802300	T	C	Cause of death: hypertensive heart disease with heart failure	Diseases and traits	1.33E-08
CYP4V2	Cyp4v3	rs532291947	chr4:187134385	T	C	Cause of death: ischaemic cardiomyopathy	Diseases andtraits	1.59E-12
CYP2J2	-	rs11572277	chrl:60374467	A	G	Cause of death: heart failure, unspecified	Diseases and traits	1.05E-07
		rs41287722	chrl:60359036	A	T	Cause of death: ischaemic cardiomyopathy	Diseases and traits	1.48E-07
CYP2C8	Cyp2c8	s56553776O	chrlO:96802435	A	G	Cause of death: peripheml vascular disease, unspecified	Diseases and traits	1.57E-18
		T8188011311	chrlO:96825092	T	C	Cause of death: dilated cardiomyopathy	Diseases andtraits	6.45E-18
CYP2C9	Cyp2c9	s55315O888	chrlO:96734381	A	G	Cause of death: peripheml vascular disease, unspecified	Diseases andtraits	9.95E-30
POR	Por	T8181538359	chr7:75566011	T	G	Cause of death: peripheml vascular disease, unspecified	Diseases and traits	6.59E-25
		T8113454523	chr7:75555973	A	G	Cause of death: congestive heart failure	Diseases and traits	1.32E-06

### 3.2 Endothelial-specific deletion of POR leads to cardiac remodelling

To validate the knockout efficiency of POR, we looked at the expression of POR in different tissues (Supplementary Figure S1). In liver (which is the organ with the highest expression of POR) there was no difference in POR at the protein level between control and ecPOR^−/−^ mice. In whole aorta, one can observe a reduction in POR in ecPOR^−/−^ mice as compared to CTL. Likewise, lung endothelial cells (LECs), enriched with magnetic beads coupled with anti-CD114 antibody show that POR is reduced in ecPOR^−/−^ LECs as compared to CTL. Altogether, the results confirm that deletion of POR using Cdh5 as a driver for deletion in endothelial cells did not alter POR levels in liver. We then focused on the knockout efficiency of POR in cardiac endothelial cells after tamoxifen treatment. ECs were enriched and compared to the remaining cellular fraction by Western blot analysis (Figure 1B). Tamoxifen treatment indeed resulted in an almost complete loss of the POR signal in the endothelial fraction with little effect on the remaining cell fraction.

To obtain a first impression on the effect of ecPOR deletion on the heart, the heart to body weight ratio was determined 30 days after tamoxifen application (Figure 1C). As compared to CTL mice, ecPOR^−/−^ male mice exhibit higher heart weight to body weight ratio, suggesting that endothelial deletion of POR may result in cardiac remodelling which could potentially lead to hypertrophy. Interestingly, this effect was not observed in female mice (data not shown). Lectin staining of the hearts revealed an increase in cardiac myocyte area (Figure 1D) in ecPOR^−/−^ mice. Likewise, the diameter of cardiac myocytes in ecPOR^−/−^ mice was increased in contrast to that of CTL mice (measured from cardiac sections stained with hematoxylin/eosin, data not shown). This suggests that endothelial deletion of POR leads to a mild, yet significant cardiac remodelling under basal condition (30 days after inducing the knockout of POR by tamoxifen feeding).

### 3.3 Endothelial deletion of POR is associated with the expression of genes linked to protein synthesis in EC and altered mitochondrial function in CM

Cardiac remodelling is complex and can be caused by numerous mechanisms including oxidative stress, metabolic reprograming, inflammation and an increase in the production of extracellular matrix (Azevedo et al., 2016). In an attempt to determine the underlying cause of cardiac remodelling in ecPOR^−/−^ mice, gene expression by RNAseq was performed from cardiac endothelial cells and the remaining cardiac tissue (left ventricle) from ecPOR^−/−^ and CTL mice.

**FIGURE 2:**
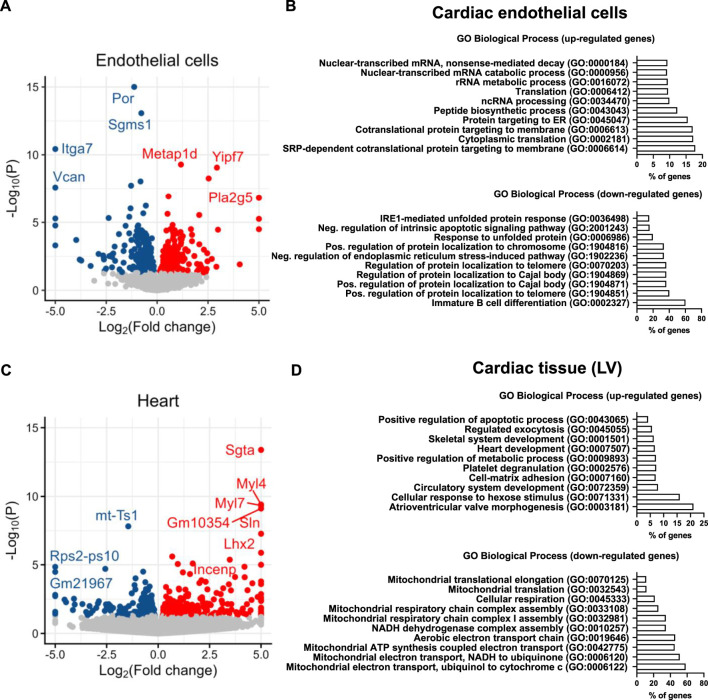
RNA-sequencing of isolated cardiac endothelial cells and left ventricle. **(A)** Volcano plot summarizing the significantly altered genes in EC. *n* = 3 (pool of 2 different animals, genes were plotted according to *p*-value). **(B)** Ontology analysis (GO Biological Process, 2021; Chen et al., 2013; Kuleshov et al., 2016; Xie et al., 2021) for the top 500 up and downregulated genes of cardiac ECs. Bars represent the percentage of overlapping genes from the top ten analyzed ontologies. **(C)** Volcano plot summarizing the significantly altered genes in the heart. *n* = 3. **(D)** Ontology analysis (GO Biological Process, 2021; Chen et al., 2013; Kuleshov et al., 2016; Xie et al., 2021) for the top 200 up and downregulated genes of the left ventricle. Bars represent the percentage of overlapping genes from the top ten analyzed ontologies. *n* = 3. *p < 0.05.

As expected, POR was among the top50 most significantly downregulated genes in cardiac ECs of ecPOR^−/−^ as compared to CTL mice (Figure 2A). Gene ontology analysis (https://maayanlab.cloud/Enrichr/enric; GO Biological Process, 2021; Chen et al., 2013; Kuleshov et al., 2016; Xie et al., 2021) suggest that processes like SRP (signal recognition particle)-dependent cotranslational protein targeting to membrane or protein targeting to ER were up-regulated (Figure 2B) upon deletion of POR. Those were associated with genes such as Yipf7 which is predicted to be involved in vesicle-mediated transport between endoplasmic reticulum and Golgi (Shaik et al., 2019) but also Rpl and Rps which are responsible for ribosome biogenesis and protein synthesis (Supplementary Table S1). Despite of an association to increased protein synthesis, the response to unfolded protein or ER stress-induced intrinsic apoptotic signalling pathway were downregulated in the ecPOR^−/−^ (Figure 2B). In turn, genes that are usually upregulated in response to stress were in fact downregulated, i.e., Hspa8, Hspa5 or Dnajb2 (Supplementary Table S1).

In the myocyte fraction of ecPOR^−/−^ mouse hearts, genes related to cardiac contractility as Myl7, Myl4 and Sln were upregulated and among the top50 regulated (Figure 2C). Genes associated with cardiac development, circulatory system development or atrioventricular valve morphogenesis were also increased ecPOR^−/−^ mice as compared to CTL (Figure 2D). Although the extent of expression change was low, hearts of ecPOR^−/−^ mice showed a gene signature for altered mitochondrial function, with the expression of genes like Nduf (complex I), Sdhd (complex II), Coq10b (coenyzme Q), Uqcr (complex III) or Cox (cytochrome c oxidase) being reduced (Supplementary Table S2).

Given that POR/CYP450 are potential sources of ROS and oxidative stress is linked to cardiac remodelling, genes associated with the term “response to oxidative stress” (GO:0006979) were analysed. There was no significant increase in the expression of enzymes which are sources of ROS, like NAPDH oxidases in neither EC nor CM. In fact, there was a minor, yet significant, decrease in the expression of Prdx2 (Peroxiredoxin-2) and Sod2 (Superoxide Dismutase) in CM (Supplementary Figure S2; Supplementary Table S3). Thus, it is unlikely that endothelial deletion of POR leads to oxidative stress given that the expression of the later enzymes is induced in response to oxidative stress (Kokoszka et al., 2001). Rather, the RNAseq data suggest that deletion of POR in endothelial cells might lead to alterations in ribosomal biogenesis and protein synthesis without inducing stress response. Such changes could yet potentially affect cardiac contractility.

### 3.4 Endothelial deletion of POR does not lead to major changes in the cardiac metabolome

**FIGURE 3:**
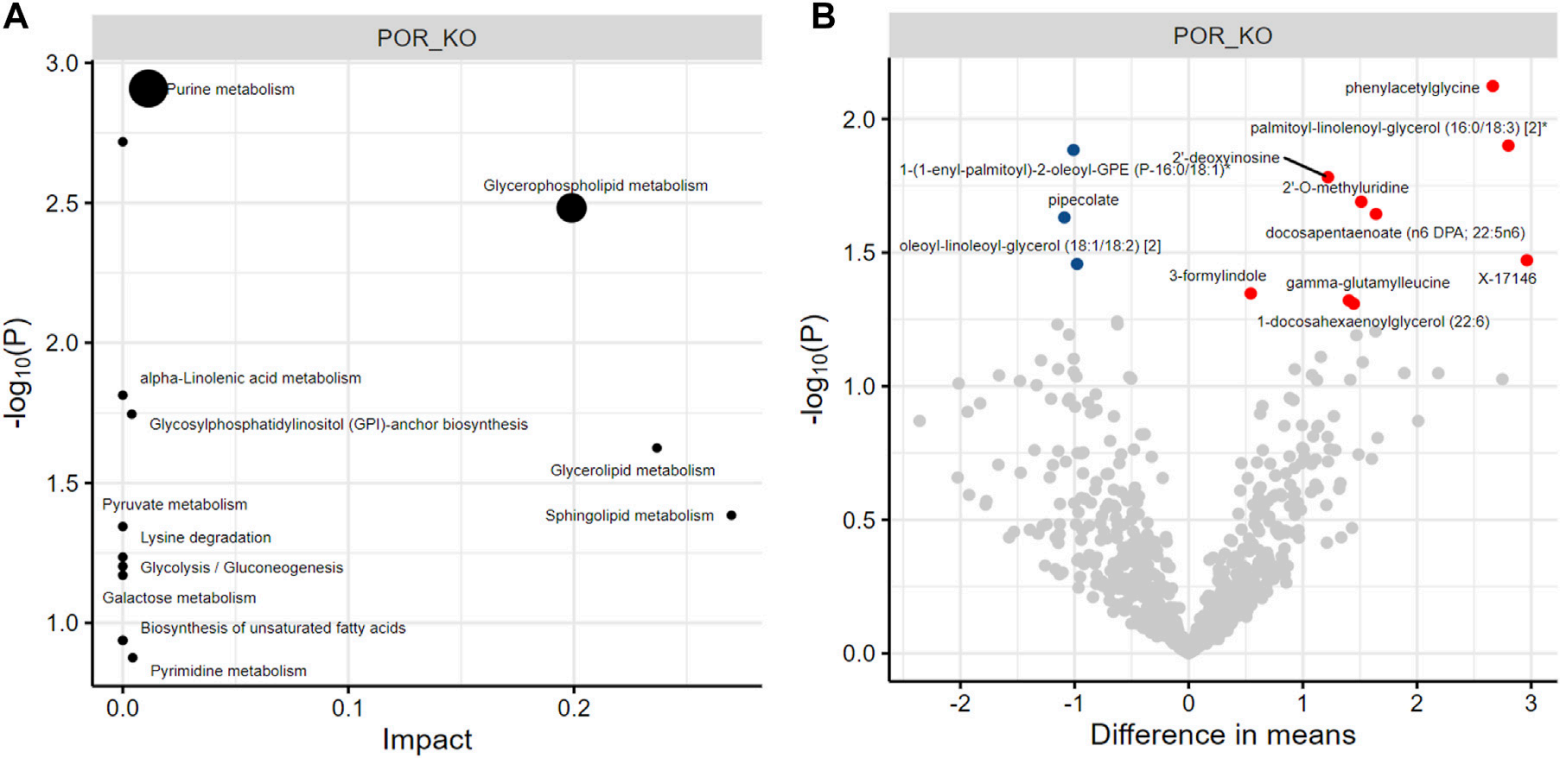
Metabolomics of cardiac tissue. Pathway enrichment **(A)** and unique metabolites **(B)** which are significantly altered in cardiac tissue of ecPOR^−/−^ mice as compared to CTL. Metabolites are represented as differential mean. Red: reduced and blue: increased in cardiac tissue of ecPOR^−/−^ mice. *n* = 7.

To investigate whether the POR/CYP450 system is involved in the ω-hydroxylation of fatty acids (through the CYP4 family) which are taken up from the blood and have to trans-pass the endothelial barrier to serve as source of energy for cardiac myocyte *via* β-oxidation, an untargeted metabolomics was performed from whole cardiac tissue. Such untargeted analysis of fatty acids, lipids of the eicosanoid class, but also of other metabolic pathways have not yet been yet explored in the context of POR/CYP450. Out of 778 metabolites identified, only 12 showed minor but significant changes in ecPOR^−/−^ hearts as compared to CTL. Of those, one is chemically unnamed (X-17146), and the others belong to purine and glycerophospholipid metabolism (Figure 3; Supplementary Table S4), which cannot be explained by the direct enzymatic function of POR. All in all changes in cardiac metabolites upon deletion of endothelial POR were minor and not direction giving. Moreover, there were no changes in the expression of genes (Myh6 and Glut4) (Supplementary Figure S3) linked to metabolic reprogramming of the heart (Jabs et al., 2018). Thus, a major metabolic function for endothelial POR/CYP450 for the heart is unlikely.

### 3.5 Endothelial deletion of POR accelerates development of heart failure in a model of pressure overload

**FIGURE 4:**
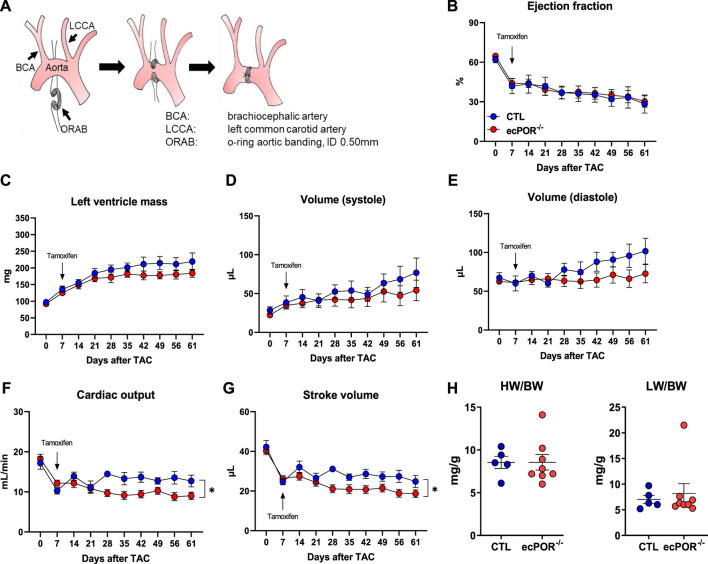
Endothelial deletion of POR accelerates heart failure in a model of pressure overload. **(A)** Schematic representation of the TAC (transverse aortic constriction) using O-ring aortic banding (ORAB; ID: 0.50 mm). **(B–G)** Cardiac parameters measured by echocardiography as indicated at baseline (0), 7, 14, 21, 28, 35, 42, 49, 56 and 61 days post TAC surgery. n ≥ 5, *p < 0.05 ecPOR^−/−^ as compared to CTL mice, Two-Way ANOVA for repeated mesurements. **(H)** Ratio of heart weight (HW) and lung weight (LW) to body weight.

To evaluate if the cardiac remodelling induced by endothelial deletion of POR had a functional consequence, CTL and ecPOR^−/−^ mice were subjected to TAC (transverse aortic constriction) as the most widely used pressure-overload model (Rockman et al., 1991) and subsequently studied by echocardiography before and after deletion of POR. The constriction was made with O-rings (Figure 4A) with an inner diameter of 0.50 mm (Melleby et al., 2018) and echocardiography was performed before the surgery and at days 7, 14, 21, 28, 35, 42, 49, 56 and 61 post surgery. TAC reduced the ejection fraction from ∼64% (day 0) to 43% at day 7. Then, the endothelial knockout of POR was induced by i.p. injection of tamoxifen. The ejection fraction progressively declined over time, with no differences between the genotypes (Figure 4B). Conversely, left ventricle mass, cardiac systolic and diastolic volumes increased in the course of TAC, however, also with no significant difference between the two groups (Figures 4C–E). In contrast, cardiac output and stroke volume were significantly reduced upon deletion of POR (Figures 4F,G). The ratios of heart weight or lung weight to body weight between the two genotypes were similar (Figure 4H).

## 4 Discussion

In the present study, an endothelial-specific, tamoxifen-inducible knockout mouse of POR was utilized to inactivate all microsomal CYP450 enzymes and investigate the cardiac consequences. This approach overcomes the frequent limitations of the CYP450 research such as: lack of specific inhibitors, compensatory expression and redundant function among the isoenzymes.

The cardiac expression of CYP450 enzymes was to some extent cell-specific. In endothelial cells, the CYP2 and CYP4 families showed the highest expression and human SNPs in members of these families are associated with cardiovascular diseases. The high expression of isoenzymes of the CYP2 family is in line with their known function in the production of EETs from arachidonic acid. This class of fatty acids is well known to be protective in the heart (Lu et al., 2001; Xiao et al., 2004; Cazade et al., 2014) for example, in myocardial ischemia/reperfusion (Gross et al., 2007; Oni-Orisan et al., 2014). Activation of K_ATP_ channels by 11,12-EET and inhibition of T-type calcium channels by 5,6-EET result in membrane repolarization, whereas 11,12-EET and 14,15-EET reduces the infarct size in rat hearts (Gross et al., 2007; Cazade et al., 2014; Oni-Orisan et al., 2014). In a TAC model, cardiac function could be restored by CYP2J2 overexpression (Edin et al., 2011) and changes in expression of CYP450 enzymes and their associated metabolites contribute to the initiation of cardiac hypertrophy (Althurwi et al., 2015). However, most of these studies are based on cardiac myocyte-specific CYP2J2 overexpression, EETs administrated by injection, knockout mouse model of soluble epoxide hydrolase (sEH) or pharmacological inhibition of sEH (Gross et al., 2007; Oni-Orisan et al., 2014). The ecPOR^−/−^ model adds insights into the specific endothelial contribution of metabolites of POR/CYP450 for cardiac function. The endothelial inactivation of CYP450 enzymes showed cardiac remodelling at basal conditions as observed by an increase in the heart to body weight ratio and of the area of cardiac myocytes in ecPOR^−/−^ mice. As the gene ontology suggested changes in the mitochondrial functional two hypotheses were raised: i. POR/CYP450 could be involved in the ω-hydroxylation of fatty acids (through the CYP4 family) which are taken up from the blood and have to trans-pass the endothelial barrier to serve as source of energy for cardiac myocyte *via* β-oxidation; ii. cardiac alterations in ecPOR^−/−^ result from the lack of endothelial EETs, which contribute to normal vascular tone and are cardioprotective molecules. However, the RNAseq of cardiac endothelial cells and cardiac tissue along with metabolomics, as two screening and unbiased approaches, did not support a metabolic role of POR/CYP450 in terms of fatty acid oxidation (ω-hydroxilation to provide carbons for β-oxidation (Hardwick, 2008)). In addition, there was no evident increase in ROS production nor oxidative stress upon deletion of endothelial POR. Rather, the reduction in cardiac output and stroke volume observed in ecPOR^−/−^ mice under TAC suggests an overall increased vascular stiffness in these mice. This consideration is supported by our previous finding that vascular EETs are reduced in ecPOR^−/−^ mice and their carotid arteries show a significantly lower diameter as compared to CTL mice (Malacarne et al., 2022).

Interestingly, endothelial deletion of POR affected the expression of genes essential for vesicle trafficking and ribosomal assembly, pointing to a function in protein synthesis and dynamics (Schwarz and Blower, 2016). As POR is localized in the endoplasmic reticulum (Barnaba et al., 2017) these results may suggest a role of POR in the regulation of membrane protein synthesis and secretion. Genes related to ER stress were downregulated, suggesting that the ER folding capacity is not exceeded. This could be a result of a compensatory mechanism. However, how an increase in endothelial genes impacts on cardiac myocyte gene expression remains unclear. Genes related to cardiac contractility were upregulated such as Myl4, Myl7 and Sln. Although it has been shown that the atrial light chain-1 (ALC1, protein coded by Myl4) is expressed in the atria, reexpression of ALC1 in the ventricle of patients with hypertrophic cardiomyopathy has been reported (Schaub et al., 1984) and improved contractility in ventricle reexpressing ALC1 is seen as an adaptive response during hypertrophy (Schaub et al., 1998).

In conclusion, the present study demonstrates that loss of endothelial POR/CYP450 function leads to cardiac remodelling.

## Data Availability

The datasets presented in this study can be found in online repositories. The names of the repository/repositories and accession number(s) can be found below: https://www.ncbi.nlm.nih.gov/, GSE214776.

## Abbreviations

AA, Arachidonic acid; CM, Cardiac myocyte; CO, Cardiac output; CTL, Control; CYP450, Cytochrome P450; EC, Endothelial cell; ecPOR^−/−^, endothelial cell-specific, tamoxifen inducible knockout mice of POR; EET, Epoxyeicosatrienoic acid; ER, Endoplasmic reticulum; FACS, Fluorescence activated cell sorting; LV, Left ventricle; ORAB, O-ring aortic banding; POR, Cytochrome P450 reductase; TAC, Transverse aortic constriction.

## References

[B1] Al-Khelaifi F. Diboun I. Donati F. Botrè F. Alsayrafi M. Georgakopoulos C. (2018). A pilot study comparing the metabolic profiles of elite-level athletes from different sporting disciplines. Sports Med. Open 4 (1), 2. 10.1186/s40798-017-0114-z 29305667 PMC5756230

[B2] Althurwi H. N. Maayah Z. H. Elshenawy O. H. El-Kadi A. O. S. (2015). Early changes in cytochrome P450s and their associated arachidonic acid metabolites play a crucial role in the initiation of cardiac hypertrophy induced by isoproterenol. Drug Metab. Dispos. 43 (8), 1254–1266. 10.1124/dmd.115.063776 26033621

[B3] Andrews S. (2010). FastQC: A quality control tool for high throughput sequence data. Available at: http://www.bioinformatics.babraham.ac.uk/projects/fastqc .

[B4] Azevedo P. S. Polegato B. F. Minicucci M. F. Paiva S. A. R. Zornoff L. A. M. (2016). Cardiac remodeling: Concepts, clinical impact, pathophysiological mechanisms and pharmacologic treatment. Arq. Bras. Cardiol. 106 (1), 62–69. 10.5935/abc.20160005 26647721 PMC4728597

[B5] Barnaba C. Martinez M. J. Taylor E. Barden A. O. Brozik J. A. (2017). Single-protein tracking reveals that NADPH mediates the insertion of cytochrome P450 reductase into a biomimetic of the endoplasmic reticulum. J. Am. Chem. Soc. 139 (15), 5420–5430. 10.1021/jacs.7b00663 28347139

[B6] Bridgewater B. R. (2014). High resolution mass spectrometry improves data quantity and quality as compared to unit mass resolution mass spectrometry in high-throughput profiling metabolomics. Metabolomics. 04 (02). 10.4172/2153-0769.1000132

[B7] Cazade M. Bidaud I. Hansen P. B. Lory P. Chemin J. (2014). 5, 6-EET potently inhibits T-type calcium channels: Implication in the regulation of the vascular tone. Pflugers Arch. 466 (9), 1759–1768. 10.1007/s00424-013-1411-0 24327205

[B8] Chaudhary K. R. Batchu S. N. Seubert J. M. (2009). Cytochrome P450 enzymes and the heart. IUBMB life 61 (10), 954–960. 10.1002/iub.241 19787709

[B50] Chen E. Y. Tan C. M. Kou Y. (2013). Enrichr: Interactive and collaborative HTML5 gene list enrichment analysis tool. BMC Bioinform. 14, 128. 10.1186/1471-2105-14-128 PMC3637064 23586463

[B9] Corey D. Anne M. Dai H. Kay A. (2012). “Software techniques for enabling high-throughput analysis of metabolomic datasets,” in Metabolomics. [Erscheinungsort nicht ermittelbar]. Editor Roessner U. (InTech).

[B10] Edin M. L. Wang Z. Bradbury J. A. Graves J. P. Lih F. B. Degraff L. M. (2011). Endothelial expression of human cytochrome P450 epoxygenase CYP2C8 increases susceptibility to ischemia-reperfusion injury in isolated mouse heart. FASEB J. 25 (10), 3436–3447. 10.1096/fj.11-188300 21697548 PMC3177568

[B11] Evans A. M. DeHaven C. D. Barrett T. Mitchell M. Milgram E. (2009). Integrated, nontargeted ultrahigh performance liquid chromatography/electrospray ionization tandem mass spectrometry platform for the identification and relative quantification of the small-molecule complement of biological systems. Anal. Chem. 81 (16), 6656–6667. 10.1021/ac901536h 19624122

[B12] Fleming I. (2014). The pharmacology of the cytochrome P450 epoxygenase/soluble epoxide hydrolase axis in the vasculature and cardiovascular disease. Pharmacol. Rev. 66 (4), 1106–1140. 10.1124/pr.113.007781 25244930

[B13] Fleming I. (2001). Cytochrome p450 and vascular homeostasis. Circ. Res. 89 (9), 753–762. 10.1161/hh2101.099268 11679404

[B14] Frömel T. Naeem Z. Pirzeh L. Fleming I. (2022). Cytochrome P450-derived fatty acid epoxides and diols in angiogenesis and stem cell biology. Pharmacol. Ther. 234, 108049. 10.1016/j.pharmthera.2021.108049 34848204

[B53] GO Biological Process (2021). Available at: https://maayanlab.cloud/Enrichr/ .

[B15] Gross G. J. Hsu A. Falck J. R. Nithipatikom K. (2007). Mechanisms by which epoxyeicosatrienoic acids (EETs) elicit cardioprotection in rat hearts. J. Mol. Cell. Cardiol. 42 (3), 687–691. 10.1016/j.yjmcc.2006.11.020 17217955 PMC1876789

[B16] Hardwick J. P. (2008). Cytochrome P450 omega hydroxylase (CYP4) function in fatty acid metabolism and metabolic diseases. Biochem. Pharmacol. 75 (12), 2263–2275. 10.1016/j.bcp.2008.03.004 18433732

[B17] Hinkel R. Ramanujam D. Kaczmarek V. Howe A. Klett K. Beck C. (2020). AntimiR-21 prevents myocardial dysfunction in a pig model of ischemia/reperfusion injury. J. Am. Coll. Cardiol. 75 (15), 1788–1800. 10.1016/j.jacc.2020.02.041 32299591

[B18] Howe K. L. Contreras-Moreira B. de Silva N. Maslen G. Akanni W. Allen J. (2020). Ensembl Genomes 2020-enabling non-vertebrate genomic research. Nucleic Acids Res. 48 (D1), D689–D695. 10.1093/nar/gkz890 31598706 PMC6943047

[B19] Hu J. Dziumbla S. Lin J. Bibli S-I. Zukunft S. de Mos J. (2017). Inhibition of soluble epoxide hydrolase prevents diabetic retinopathy. Nature 552 (7684), 248–252. 10.1038/nature25013 29211719 PMC5828869

[B20] Jabs M. Rose A. J. Lehmann L. H. Taylor J. Moll I. Sijmonsma T. P. (2018). Inhibition of endothelial notch signaling impairs fatty acid transport and leads to metabolic and vascular remodeling of the adult heart. Circulation 137 (24), 2592–2608. 10.1161/CIRCULATIONAHA.117.029733 29353241

[B21] Jensen S. B. Thodberg S. Parween S. Moses M. E. Hansen C. C. Thomsen J. (2021). Biased cytochrome P450-mediated metabolism via small-molecule ligands binding P450 oxidoreductase. Nat. Commun. 12 (1), 2260. 10.1038/s41467-021-22562-w 33859207 PMC8050233

[B22] Kamat M. A. Blackshaw J. A. Young R. Surendran P. Burgess S. Danesh J. (2019). PhenoScanner V2: An expanded tool for searching human genotype-phenotype associations. Bioinforma. Oxf. Engl. 35 (22), 4851–4853. 10.1093/bioinformatics/btz469 PMC6853652 31233103

[B23] Kivelä R. Hemanthakumar K. A. Vaparanta K. Robciuc M. Izumiya Y. Kidoya H. (2019). Endothelial cells regulate physiological cardiomyocyte growth via VEGFR2-mediated paracrine signaling. Circulation 139 (22), 2570–2584. 10.1161/CIRCULATIONAHA.118.036099 30922063 PMC6553980

[B24] Kokoszka J. E. Coskun P. Esposito L. A. Wallace D. C. (2001). Increased mitochondrial oxidative stress in the Sod2 (+/-) mouse results in the age-related decline of mitochondrial function culminating in increased apoptosis. Proc. Natl. Acad. Sci. U. S. A. 98 (5), 2278–2283. 10.1073/pnas.051627098 11226230 PMC30129

[B51] Kuleshov M. V. Jones M. R. Rouillard A. D. Fernandez N. F. Duan Q. Wang Z. (2016). Enrichr: A comprehensive gene set enrichment analysis web server 2016 update. Nucleic Acids Res. 8 (44), (W1):W90-7. 10.1093/nar/gkw377 PMC4987924 27141961

[B25] Love M. I. Huber W. Anders S. (2014). Moderated estimation of fold change and dispersion for RNA-seq data with DESeq2. Genome Biol. 15 (12), 550. 10.1186/s13059-014-0550-8 25516281 PMC4302049

[B26] Lu T. Hoshi T. Weintraub N. L. Spector A. A. Lee H. C. (2001). Activation of ATP-sensitive K(+) channels by epoxyeicosatrienoic acids in rat cardiac ventricular myocytes. J. Physiol. 537 (3), 811–827. 10.1111/j.1469-7793.2001.00811.x 11744757 PMC2278996

[B27] Malacarne P. F. Ratiu C. Gajos-Draus A. Müller N. Lopez M. Pflüger-Müller B. (2022). Loss of endothelial cytochrome P450 reductase induces vascular dysfunction in mice. Hypertension 79 (6), 1216–1226. 10.1161/HYPERTENSIONAHA.121.18752 35354305

[B28] Melleby A. O. Romaine A. Aronsen J. M. Veras I. Zhang L. Sjaastad I. (2018). Romaine, Andreas; Aronsen, Jan Magnus; Veras, Ioanni; Zhang, Lili; Sjaastad, Ivar et alA novel method for high precision aortic constriction that allows for generation of specific cardiac phenotypes in mice. Cardiovasc. Res. 114 (12), 1680–1690. 10.1093/cvr/cvy141 29878127

[B29] Oni-Orisan A. Alsaleh N. Lee C. R. Seubert J. M. (2014). Epoxyeicosatrienoic acids and cardioprotection: The road to translation. J. Mol. Cell. Cardiol. 74, 199–208. 10.1016/j.yjmcc.2014.05.016 24893205 PMC4115045

[B30] Pandey A. V. Flück C. E. (2013). NADPH P450 oxidoreductase: Structure, function, and pathology of diseases. Pharmacol. Ther. 138 (2), 229–254. 10.1016/j.pharmthera.2013.01.010 23353702

[B31] Patro R. Duggal G. Love M. I. Irizarry R. A. Kingsford C. (2017). Salmon provides fast and bias-aware quantification of transcript expression. Nat. Methods 14 (4), 417–419. 10.1038/nmeth.4197 28263959 PMC5600148

[B32] Perbellini F. Watson S. A. Bardi I. Terracciano C. M. (2018). Heterocellularity and cellular cross-talk in the cardiovascular system. Front. Cardiovasc. Med. 5, 143. 10.3389/fcvm.2018.00143 30443550 PMC6221907

[B33] R Core Team (2015). R: A language and environment for statistical computing. Vienna, Austria: R Foundation for Statistical Computing.

[B34] Ramanujam D. Sassi Y. Laggerbauer B. Engelhardt S. (2016). Viral vector-based targeting of miR-21 in cardiac nonmyocyte cells reduces pathologic remodeling of the heart molecular therapy : The. Mol. Ther. 24 (11), 1939–1948. 10.1038/mt.2016.166 27545313 PMC5154480

[B35] Ramanujam D. Schön A. P. Beck C. Vaccarello P. Felician G. Dueck A. (2021). MicroRNA-21-Dependent macrophage-to-fibroblast signaling determines the cardiac response to pressure overload. Circulation 143 (15), 1513–1525. 10.1161/CIRCULATIONAHA.120.050682 33550817 PMC8032214

[B36] Rockman H. A. Ross R. S. Harris A. N. Knowlton K. U. Steinhelper M. E. Field L. J. (1991). Segregation of atrial-specific and inducible expression of an atrial natriuretic factor transgene in an *in vivo* murine model of cardiac hypertrophy. Proc. Natl. Acad. Sci. U. S. A. 88 (18), 8277–8281. 10.1073/pnas.88.18.8277 1832775 PMC52490

[B37] Schaub M. C. Hefti M. A. Zuellig R. A. Morano I. (1998). Modulation of contractility in human cardiac hypertrophy by myosin essential light chain isoforms. Cardiovasc. Res. 37 (2), 381–404. 10.1016/S0008-6363(97)00258-7 9614495

[B38] Schaub M. C. Tuchschmid C. R. Srihari T. Hirzel H. O. (1984). Myosin isoenzymes in human hypertrophic hearts. Shift in atrial myosin heavy chains and in ventricular myosin light chains. Eur. Heart J.F 5, 85–93. 10.1093/eurheartj/5.suppl_f.85 6241906

[B39] Schwarz D. S. Blower M. D. (2016). The endoplasmic reticulum: Structure, function and response to cellular signaling. Cell. Mol. Life Sci. 73 (1), 79–94. 10.1007/s00018-015-2052-6 26433683 PMC4700099

[B40] Shaik S. Pandey H. Thirumalasetti S. K. Nakamura N. (2019). Characteristics and functions of the Yip1 domain family (YIPF), multi-span transmembrane proteins mainly localized to the Golgi apparatus. Front. Cell Dev. Biol. 7, 130. 10.3389/fcell.2019.00130 31417902 PMC6682643

[B41] Staley J. R. Blackshaw J. Kamat M. A. Ellis S. Surendran P. Sun B. B. (2016). PhenoScanner: A database of human genotype-phenotype associations. Bioinforma. Oxf. Engl. 32 (20), 3207–3209. 10.1093/bioinformatics/btw373 PMC5048068 27318201

[B42] Sun D. Ojaimi C. Wu H. Kaley G. Huang A. (2012). CYP2C29 produces superoxide in response to shear stress. Microcirculation 19 (8), 696–704. 10.1111/j.1549-8719.2012.00202.x 22708815 PMC4550326

[B43] Talman V. Kivelä R. (2018). Cardiomyocyte-endothelial cell interactions in cardiac remodeling and regeneration. Front. Cardiovasc. Med. 5, 101. 10.3389/fcvm.2018.00101 30175102 PMC6108380

[B44] Wang B. Wu L. Chen J. Dong L. Chen C. Wen Z. (2021). Metabolism pathways of arachidonic acids: Mechanisms and potential therapeutic targets. Signal Transduct. Target. Ther. 6 (1), 94. 10.1038/s41392-020-00443-w 33637672 PMC7910446

[B45] Wang Y. Nakayama M. Pitulescu M. E. Schmidt T. S. Bochenek M. L. Sakakibara A. (2010). Ephrin-B2 controls VEGF-induced angiogenesis and lymphangiogenesis. Nature 465 (7297), 483–486. 10.1038/nature09002 20445537

[B46] Wu L. Gu J. Weng Y. Kluetzman K. Swiatek P. Behr M. (2003). Conditional knockout of the mouse NADPH-cytochrome p450 reductase gene. Genesis 36 (4), 177–181. 10.1002/gene.10214 12929087

[B47] Xiao Y-F. Ke Q. Seubert J. M. Bradbury J. A. Graves J. Degraff L. M. (2004). Enhancement of cardiac L-type Ca2+ currents in transgenic mice with cardiac-specific overexpression of CYP2J2. Mol. Pharmacol. 66 (6), 1607–1616. 10.1124/mol.104.004150 15361551

[B52] Xie Z. Bailey A. Kuleshov M. V. Clarke D. J. B. Evangelista J. E. Jenkins S. L. (2021). Gene set knowledge discovery with Enrichr. Curr. Protoc.. 10.1002/cpz1.90 PMC8152575 33780170

[B48] Zacchigna S. Paldino A. Falcão-Pires I. Daskalopoulos E. P. Dal Ferro M. Vodret S. (2021). Towards standardization of echocardiography for the evaluation of left ventricular function in adult rodents: A position paper of the ESC working group on myocardial function. Cardiovasc. Res. 117 (1), 43–59. 10.1093/cvr/cvaa110 32365197

[B49] Zanger U. M. Schwab M. (2013). Cytochrome P450 enzymes in drug metabolism: Regulation of gene expression, enzyme activities, and impact of genetic variation. Pharmacol. Ther. 138 (1), 103–141. 10.1016/j.pharmthera.2012.12.007 23333322

