# Supplementary figures and images for: Endothelial deletion of the cytochrome P450 reductase leads to cardiac remodelling
Source: Front Physiol. 2022 Dec 2;13:1056369. doi: 10.3389/fphys.2022.1056369 (PMC9755668; doi:10.3389/fphys.2022.1056369)

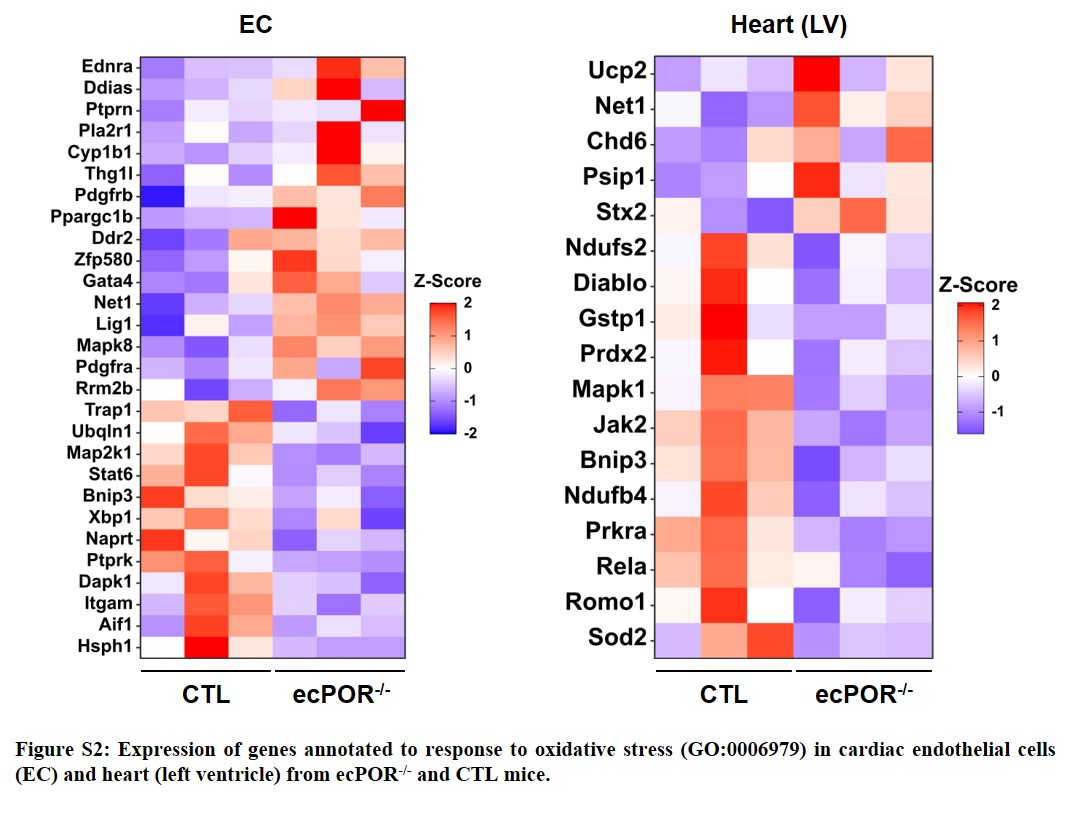

Supplement: Supplementary file 2 [file Figure2.JPEG]

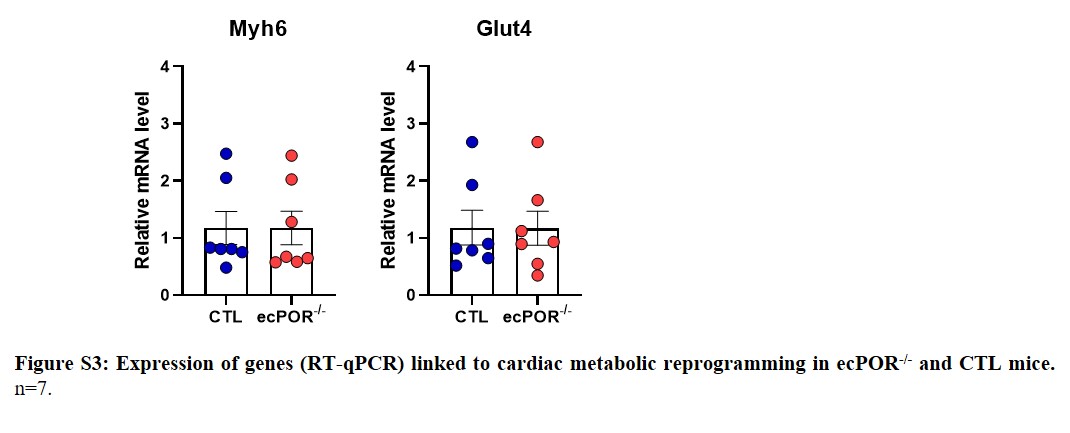

Supplement: Supplementary file 3 [file Figure3.JPEG]

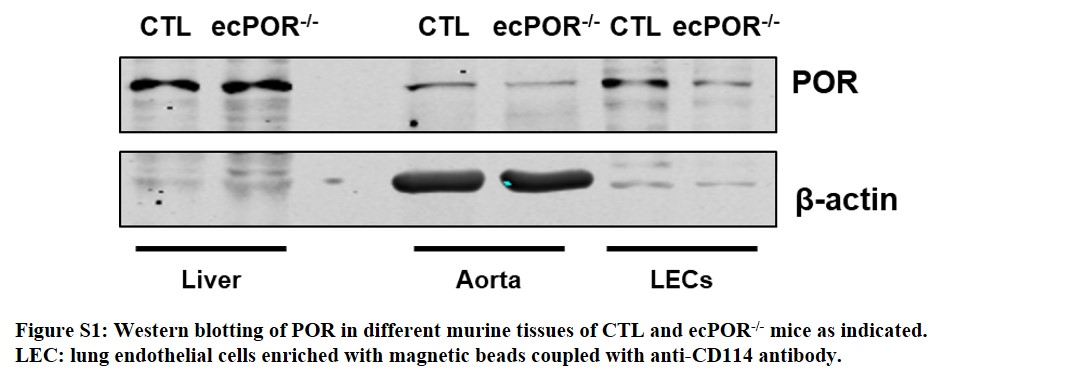

Supplement: Supplementary file 5 [file Figure1.JPEG]
